# Impact of Extremely Hot Days on Emergency Department Visits for Cardiovascular Disease among Older Adults in New York State

**DOI:** 10.3390/ijerph16122119

**Published:** 2019-06-14

**Authors:** Mengxuan Li, Benjamin A. Shaw, Wangjian Zhang, Elizabeth Vásquez, Shao Lin

**Affiliations:** 1 Department of Epidemiology and Biostatistics, University at Albany School of Public Health, Rensselaer, NY 12144, USA; dream_li@hotmail.com (M.L.); evasquez2@albany.edu (E.V.); 2 Department of Health Policy, Management and Behavior, University at Albany School of Public Health, Rensselaer, NY 12144, USA; bashaw@albany.edu; 3 Department of Environmental Health Sciences, University at Albany School of Public Health, Rensselaer, NY 12144, USA; wzhang27@albany.edu

**Keywords:** hot day, cardiovascular diseases, summer and transitional months, older adults, emergency department visits

## Abstract

Prior studies have reported the impact of ambient heat exposure on heat-related illnesses and mortality in summer, but few have assessed its effect on cardiovascular diseases (CVD) morbidity, and the association difference by demographics and season. This study examined how extremely hot days affected CVD-related emergency department (ED) visits among older adults from 2005–2013 in New York State. A time-stratified case-crossover design was used to assess the heat–CVD association in summer and transitional months (April–May and September–October). Daily mean temperature >95th percentile of regional monthly mean temperature was defined as an extremely hot day. Extremely hot days were found to be significantly associated with increased risk of CVD-related ED visits at lag day 5 (OR: 1.02, 95% CI: 1.01–1.04) and lag day 6 (OR: 1.01, 95% CI: 1.00–1.03) among older adults in summer after controlling for PM_2.5_ concentration, relative humidity, and barometric pressure. Specifically, there was a 7% increased risk of ischemic heart disease on the day of extreme heat, and increased risks of hypertension (4%) and cardiac dysrhythmias (6%) occurred on lag days 5 and 6, respectively. We also observed large geographic variations in the heat–CVD associations.

## 1. Background

Cardiovascular disease (CVD) is a leading cause of death and a major cause of hospital admission around the world. In the United States (US), it has been reported that about 610,000 deaths, 70.7 million outpatient physician office visits, and 4.5 million emergency department (ED) visits are attributable to CVD every year [1]. The estimated health care costs and the value of lost productivity due to CVD is more than $400 billion per year [2,3].

Older adults tend to be the most vulnerable population to CVD, with about two-thirds of CVD deaths occurring among the elderly population [4]. In New York State (NYS), an annual official report indicated that the main significant increase in CVD mortality starts at age 65 and older [5]. Therefore, the risk of CVD among older adults is an important public health concern. 

Older adults are also more susceptible to heat stress because they are more likely to have chronic medical conditions that alter the normal body response to heat. In addition, older adults are more likely to take prescription medicines that affect the body’s ability to control its temperature or sweat [6]. A study examining the association between temperature and mortality in the US found that older adults are particularly vulnerable to heat-related death because of their impaired heat-adaption capabilities [5]. Another study conducted in China estimated that about 17.1% of CVD mortality in 15 megacities could be attributed to the impact of ambient temperature [7]. Song et al. (2017), in a literature review, indicated that heat exposure was not found to have a major impact on cardiovascular morbidity among the general population [8]; however, Bunker et al. (2016) reported a significant heat effect on cardiovascular morbidity among the elderly population [9]. The heat effect on morbidity may be more serious among the elderly population. However, significant gaps remain in our understanding of the association between extreme heat and CVD morbidity, especially ambient heat’s effect on urgent care use such as emergency department (ED) visits, and CVD subtypes among older adults. 

The association between extreme heat and public health outcomes is complicated due to several factors, such as the different lag effects of extreme heat on different health outcomes, and possible interactions between exposure to extreme heat and other seasonal, geographic or demographic variables. For example, previous studies have suggested a higher population health risk during transitional months as compared with typical summer and winter [10], while others have suggested that residents in certain areas (coastal area or mountainous area) [11], or with certain population characteristics (poor ability to acclimate to heat) [12], were more vulnerable to environmental hazards than other groups. However, there is a scarcity of information about how these factors potentially modify the association between extremely hot days and CVD ED among older adults. 

To address these knowledge gaps, this study aims to: (1) assess the association between extremely hot days and total and specific CVD ED visits among older adults in summer and transitional months in NYS, while considering lag effects; and (2) evaluate whether the association is modified by geographic or demographic variables and disease subtypes. 

## 2. Methods

### 2.1. Study Design and Health Outcomes

We employed a bidirectional, time-stratified case-crossover design, with the patient ED visit date as the case day. Control days (without cases) were selected on the same day of the week and within the same admission month as case days [13]. Each case day was matched with at least three control days. By comparing differences in exposure between the case day and control days, we were able to evaluate whether the exposure was associated with ED visit for CVD. We selected the case-crossover design as this commonly used method can assess the acute effect of extreme heat on health endpoints, and the impact of heat on CVD seemed to occur within one week based on prior literature [14,15,16]. Another advantage of the case-crossover design is that the effects of some inherited factors which are not available in our data, such as family history of CVD, CVD treatment, family income, smoking, alcohol drinking, etc., are automatically controlled by this design.

The study population consisted of adults aged 65 years and older in NYS. We obtained ED visits data for CVDs across NYS for the months of April to October during the years 2005 to 2013 from the NYS Department of Health’s Statewide Planning and Research Cooperation System (SPARCS). The SPARCS data included International Classification of Disease 9th version (ICD9) codes [17], age, gender, race, ethnicity, residential address, and date of admission of at least 95% of all acute-care inpatient and outpatient cases in NYS [18]. Based on the previous literature [14,19,20], the ICD9 codes for the following principal diagnosis were extracted from SPARCS: chronic rheumatic heart diseases (393–396), hypertension (401–405), ischemic heart diseases (410–414), cardiac dysrhythmias (427), congestive heart failure (428), and cerebrovascular diseases (430–434, 436–438). However, since chronic rheumatic heart disease, congestive heart failure, and cerebrovascular disease had small sample sizes, we merged all of these diseases together into “other CVDs” group as defined in previous literature [14]. This study received approval from the NYS Department of Health (DOH) Institutional Review Board (IRB) and approval from the NYSDOH Data Governance Committee (1509-01 A) for access to the SPARCS data.

### 2.2. Weather Data and Exposure Definition 

Daily mean temperature, dew point temperature, and barometric pressure values were retrieved from the National Climatic Data Center (NCDC), while daily mean PM_2.5_ (particular matter less than 2.5 μm in aerodynamic diameter) was collected from the United States Environmental Protection Agency (EPA) for the study period (2005–2013). Summer was defined as the period between June and August. The ambient heat exposure in this study was dichotomized using the 95th percentile of daily mean temperature per that specific month in each region based on prior research [21,22]. Since our previous studies found a stronger health effect from extreme weather in transitional months than in summer or winter in NYS [22,23], we extended the summer period to cover transitional months April–May and September–October to represent transitional months in spring and fall, respectively. The daily relative humidity (RH) was calculated using the dew point temperature and daily mean temperature, with the following formula:

RH = EXP (log (10) *(7.5*D/(237.7 + D)−7.5*T/(237.7 + T))),

where the temperature is in degrees Fahrenheit and daily relative humidity is a percentage. According to previous research [14], relative humidity and barometric pressure are associated with CVD hospitalization among older adults, and these factors were therefore considered as potential confounders in the current study. Weather records from 69 NCDC monitoring sites and air pollutant data from 46 EPA monitoring sites were included in our study. Based on the coordinates of these sites, we assigned them to 14 weather regions in NYS which have been described in previous studies [24,25,26]. During certain days of the study period, we observed that some regions did not have monitoring stations, and some stations did not have daily NCDC data or EPA data. To address these issues, we used two approaches to handle missing values: (1) In the absence of a station in the weather region, we assigned values from the nearest station; and (2) where daily climate data were not recorded during a certain period, we applied a 7-day moving average value to replace missing values (e.g., barometric pressure and PM_2.5_ value in climate region). We used MapInfo Pro v12.5 (MapInfo Corp., Troy, New York, USA) to geocode all older adult patients’ addresses and assigned them to 14 weather regions before linking them to the weather data for that region.

### 2.3. Statistical Analysis

We assessed the association between extremely hot days and ED visits for CVD among older adults by using conditional logistic regression after controlling for daily PM_2.5_ concentration values, daily relative humidity, and daily barometric pressure, and evaluated lagged effects up to 6 days prior to ED visit day. The same analyses and methods were applied to examine the heat–CVD associations in each CVD subtype. To evaluate the potential influence of cut-off point selection in defining extreme heat indicators, we also performed analyses using 90th and 97th percentiles. All analyses were performed using the PHREG procedure in SAS, version 9.4, statistical software (SAS Institute, Inc., Cary, NC, USA). 

## 3. Results

### 3.1. Characteristics of Population and Extremely Hot Days

Our analytic sample consisted of 416,707 older adult patients who visited an emergency department due to CVD in NYS from April to October from 2005–2013. Of these, hypertensive disease (220,058 cases, 53%) comprised the highest proportion among all the diagnoses (Table 1). The mean daily temperature was 53.8 °F (12.1 °C) in spring months, 70.6 °F (21.4 °C) in summer, and 58.2 °F (14.6 °C) in fall months. The 95th percentile daily mean temperature for the study period ranged from 83.4 °F (28.6 °C) in Staten Island to 62.3 °F (16.8 °C) in the Adirondack and North regions (Table 2). During the entire study period, 2,023 extremely hot days occurred in the 14 weather regions, with every region having at least one heat day per month, and some regions (e.g., New York City) having 3–4 extremely hot days in summer, in some cases on consecutive days.

### 3.2. Association between Extremely Hot Days and Older Adults ED Visits

**Figure 1:**
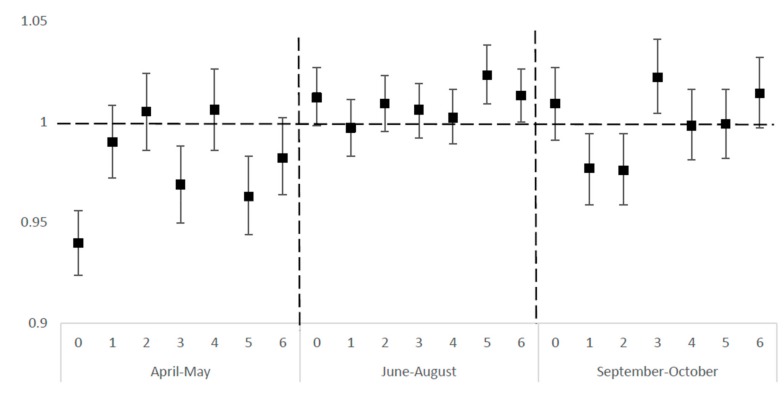
Odds ratios for association between extremely hot days * in lagged days and emergency department (ED) elderly visits for cardiovascular disease (CVD) in New York State (NYS), April–October 2005–2013. * Extremely hot day is defined as daily mean temperature >95th percentile of regional monthly mean temperature.

Figure 1 shows the odds ratios for the association between extreme heat and overall ED visits for CVDs in summer and transitional months in NYS (2005–2013), and the lagged day effects. After controlling for PM_2.5_ concentration, relative humidity, and barometric pressure, extremely hot days were significantly associated with increased odds of older adults ED visits at lag day 5 (OR: 1.023, CI: 1.009–1.038) and lag day 6 (OR: 1.013, CI: 1.001–1.026) in summer, and at lag day 3 (OR: 1.022, CI: 1.004–1.041) in the fall. In spring months, extremely hot days showed a protective effect at lag day 0 (OR: 0.940, CI: 0.924–0.956), lag day 3 (OR: 0.969, CI: 0.950–0.988), and lag day 5 (OR: 0.963, CI: 0.944–0.983). Similar effects appeared at lag day 1 (OR: 0.977, CI: 0959–0.994) and lag day 2 (OR: 0.976, CI: 0.959–0.994) in the fall.

We did not find any clear dose–response relationship between the frequency or duration of extremely hot days and the risk of CVDs ED visits among the elderly. The trend analyses were not significant. (Results shown in Appendix A Table A1 and Table A2).

### 3.3. Impact of Extremely Hot Days on Risk of Specific CVD Category

**Figure 2:**
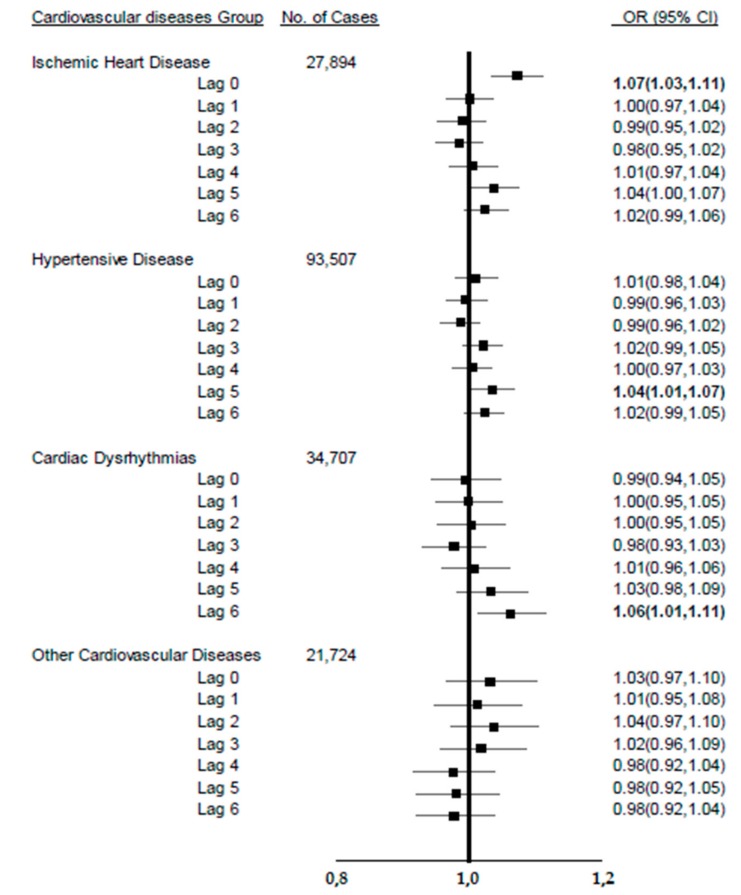
The association between extremely hot days (>95%) in lagged days and emergency department visits for four cardiovascular diseases diagnostic categories in New York State (NYS), summer (June–August), 2005–2013.

Figure 2 shows the associations between exposure to hot days and ED visits due to different CVD subtypes in the summer. Statistically significant increased associations between extreme heat and CVD were found for ischemic heart disease in the same day of an extremely hot day (OR: 1.07, 95% CI: 1.03–1.11). In addition, significantly increased associations were found at lag day 5 (hypertensive heart disease (HHD): OR: 1.04, CI: 1.01–1.07) and lag day 6 (cardiac dysrhythmias: OR: 1.06, CI: 1.01–1.11) in the summer.

### 3.4. Comparison of Different Heat Indicators

As shown in Table 3, we compared the CVD ED risks during extremely hot days to those of non-extremely hot days using different extreme heat indicators, i.e., the 90th, 95th, and 97th percentiles of monthly daily average temperatures according to previous studies [21,22,27]. The point estimates changed, but trends were similar under all definitions considered, with adverse and significant associations observed (Lag 6: 90th OR: 1.022, CI: 1.01–1.04; 95th OR: 1.01, CI: 1.00–1.03; 97th OR: 1.04, CI: 1.01–1.06). 

### 3.5. Stratified Analysis by Demographics on Heat–CVD

We performed stratified analyses to examine if the heat–CVD association varied by demographic groups. Because the main hot day effect appeared in summer, we mainly evaluated the association in different geographical and demographic strata in June–August. Only one group showed a statistically significant association: Compared to older adults’ ED visits in other regions, there was an 8.5% increased risk for older adults’ ED visits on hot days in Long Island (95% CI: 1.024–1.149) (Table 4).

## 4. Discussion

We observed significant associations between extremely hot days and ED visits due to CVDs among older adults during summer after controlling for PM_2.5_ concentration, relative humidity, and barometric pressure. Our overall results of 2.3% excess odds for elderly ED visits on the 5th day and 1.3% excess odds for elderly ED visits on the 6th day after their exposure to extremely hot days in summer are similar to those of other heat and morbidity studies. For example, Green et al. (2010) found that a 10 °F increase in mean apparent temperature was associated with a 3.5% increase in CVDs hospital admission [19]. Dawson et al. (2008) detected that a 1 °C (1.8 °F) increase in mean temperature during the preceding 24 hours was associated with a 2.1% increase in ischemic stroke hospital admission [28]. Giang et al. (2014) performed an analysis of the effect of temperature on CVDs hospital admission among the elderly in Vietnam and found an increased risk of CVDs admissions from lag day 5 to lag day 10 [29]. 

While we found positive associations between extremely hot days and ED visits for CVDs in summer, we found non-significant, even protective effects in transitional months. However, this finding is in fact reasonable in light of the biological mechanisms involved in temperature change and CVDs, which other studies have mentioned as well [19,20,22]. When the weather turns warmer in spring, the long-term cold-stress weakens, leading to systemic vasodilation, and blood pressure returns to the normal range. In summer, extreme high temperature may increase plasma viscosity and serum cholesterol levels, which could lead more ischemic heart diseases and cerebrovascular diseases and trigger a sudden rise in blood viscosity and cardiac output, which may cause cardiac dysrhythmias and hypertension [30]. As the temperatures in both transitional months in NYS were over 10 °F lower than that in the summer, it would be difficult to observe the heat effect in these transitional months. Springtime is a period of relief from cold temperatures, during which warmer temperatures may be better for the cardiovascular system, particularly for older adults. 

The positive effect of heat on ischemic heart diseases (IHD) was observed to be acute, with ED visits occurring on the same day of extreme heat. A systematic review of the literature indicated that an immediate elevated risk of myocardial infarction hospitalization was associated with each 1 °C ambient temperature increase (RR: 1.016, 95%CI: 1.004–1.028) [31], which is consistent with the immediate heat effect on the ischemic heart disease ER visits in our study.

We compared the CVD results from extreme heat days defined by different temperature percentiles (90th, 95th, and 97th percentiles) to determine which indicators are more sensitive to extreme heat. We found that there are no significant differences in odds ratios among these indicators and found that the excess odds for elderly ED visits on the 6th day after exposure to extremely hot days in summer is consistent across these percentiles.

Interestingly, different lag effects were found for various CVD subtypes in this study. Hypertensive heart diseases (HHD) and cardiac dysrhythmias showed delayed effects in summer—at lag day 5 and lag day 6, respectively. These lag effects are consistent with previous studies [15,25,26]. Schwartz et al. (2004) estimated the effects of temperature and humidity on CVD hospital admissions of elderly in 12 US cities, and they found that heat effects on hospital admissions mainly occur within 7 days after exposure [15]. Guo et al. (2013) found the most pronounced heat effect of temperature (RR: 1.24, 95% CI: 1.09–1.39) on IHD mortality in lag days 0–13 compared to the heat effect on lag days 0–2 when they studied ischemic heart disease mortality in five metropolis areas in China [32]. Wang et al. (2015) used time series analysis to evaluate the extreme heat effect on IHD and HHD mortalities in China [33]. They found that the deaths of IHD and HHD in Beijing showed high susceptibility to the extremely high temperature in lag days 0–14 in summer (IHD RR: 1.15,95%: 1.05–1.24; HHD RR:1.39, 95%: 1.01–1.92). Lin et al. (2009) performed an analysis of the heat effect on CVD hospital admission in New York City (NYC) and found extreme temperature resulted in increased lagged hospital admissions for CVD at lag day 1 (2.5%), lag day 2 (2.1%) and lag day 3 (3.6%) [14]. The positive associations found between extremely hot days and lagged CVD sub-category (IHD, HHD, and cardiac dysrhythmias) mortality and mobility from all these prior studies are consistent with our findings.

Some studies have found significant increases in cardiovascular mortality associated with heat in specific demographic and geographic groups, but this kind of effect on morbidity has rarely been assessed. Although neither gender- nor race-specific effect differences were shown in our study, a significantly higher risk of CVD ED visits due to extreme heat was found in Long Island in summer. A study focused on the relationship between heat and CVD hospitalization in NYC showed a similar result. Lin et al. (2009) found a 3.61% increased risk for CVD hospitalization when daily mean temperature was above the heat threshold in NYC. They also found older adults to be one of the most vulnerable groups in the study [14]. Generally, coastal areas experience lower temperatures than inland regions, so even though the 95th percentile is a low temperature, it may be higher than what the residents are used to. Thus, we see the impact of heat at lower temperatures than in inland populations. Humidity, barometric pressure, and high temperatures together amplify the effect of heat alone. We speculated that the main reason for this is that Long Island had the highest relative humidity and barometric pressure compared to NYC or upstate areas in summer. These two factors, combined with high temperature, could form an unusually humid heat wave, which may impact older adults’ health. One study from California which may support this hypothesis indicated that compared to exposure to dry heat waves in inland areas, humid heat waves in the coastal area made citizens, especially older adults, more uncomfortable [11]. Additionally, during humid heat wave periods, there will be unprecedented high temperatures during both daytime and nighttime, and with early morning being one of the highest risk times of CVDs, this may compound the risk of older adult heart attacks hospitalization. 

### Study Strengths and Limitations

This is one of few studies evaluating the association between heat and CVDs among older adults using ED data. Older people are more vulnerable to extreme temperatures, and ED visit data can provide an indication of early and acute heat-related effects. Additional strengths include controlling for personal confounders like age, gender, race, and ethnicity through the case-crossover study design and controlling for other potential confounders such as air pollution and other meteorological factors. Assessing PM_2.5_ concentrations as a potential confounder has been shown to both impact CVDs hospitalizations [34,35] and to be associated with the temperature distribution. This is the only study that evaluates heat’s impacts on specific CVD types and by summer and transitional months in New York State.

This study has some limitations which we should report. First, the weather data come from stationary monitoring stations, and we assume that all the monitoring values are similar within each weather region. There are a limited number of monitor stations, and this leads to missing data. Violations of this assumption will lead results toward the null, and thus, our results may be underestimated, but this is a limitation across similar studies using weather monitor data [20]. We minimized this bias by averaging the stations’ monitor values if there is more than one station in that region, or in cases of missing monitor values in certain days. Second, time–activity pattern information is not included in the study. Some unmeasured behavioral factors that may bias our results include air conditioner usage and the time older adults spent outdoors. Even though this information was not available in our current dataset, our study design—case-crossover—which compared cases to themselves during the study period, can minimize this bias, as the older adult’s time–activity patterns are not likely to change considerably during the study period. In addition, our use of ED data may only catch the severe CVDs, as every case invariably ends up in hospitalization data. Finally, we may be only capturing a small percent of IHD events. However, considering that most CVDs would go to urgent care or ED/hospitalization, this implies that ED visit may be a good indicator for CVD recruitment.

## 5. Conclusions

We observed significantly increased risks of CVD-related ED visits among older adults in NYS with acute and lagged extreme heat exposure in the summer. Extreme heat had immediate effects on IHD but delayed effects on hypertensive and cardiac dysrhythmia cases with five or six lag days. Older adults living in Long Island had a higher risk of ED visits due to CVDs compared to those in other regions. However, we did not find similar heat effects on CVDs in the transitional months. The results from this study provide valuable information for health policymakers and health care providers for potential education planning and targeted intervention, especially among the older adults.

## Figures and Tables

**Table 1:** Distributions of principal diagnoses for cardiovascular diseases emergency department elderly visits in New York State, April–October 2005–2013.

Disease	ICD-9 Codes	No. of Cases	Percent Distribution (%)
Total Cardiovascular diseases	393–438	416,707	100
Hypertensive disease	401–405	220,058	52.81
Ischemic heart disease	410–414	64,262	15.42
Cardiac dysrhythmias	427	81,290	19.51
Other cardiovascular disease *	393–396, 428, 430–434, 436–438	51,097	12.26

* Other diseases including chronic rheumatic heart disease, congestive heart failure, and cerebrovascular disease. ICD-9: International Classification of Disease 9th version codes.

**Table 2:** Summary statistics of daily weather conditions in transitional months and summer in New York State, 2005–2013.

Region	April–May (Spring)					June–August (Summer)					September–October (Fall)				
	Average Temperature *	95th Percentile of Daily Temperature *	Mean PM_2.5_ *	Mean RH *	Mean BP *	Average Temperature	95th Percentile of Daily Temperature	Mean PM_2.5_	Mean RH	Mean BP	Average Temperature	95th Percentile of Daily Temperature	Mean PM_2.5_	Mean RH	Mean BP
**Statewide**	53.8	67.3	7.1	61.3	995.2	70.6	78.7	10.5	69.8	994.8	58.2	69.0	7.2	71.7	997.2
NYC-LaGuardia Airport	58.0	71.8	9.5	58.6	1010.0	74.5	82.8	12.8	65.8	1009.2	62.9	73.1	8.9	66.5	1012.0
NYC- Kennedy Airport	57.3	69.0	8.6	58.9	1014.2	75.2	83.0	12.0	63.4	1013.4	64.1	73.7	8.1	63.2	1016.0
Staten Island	57.9	71.6	8.2	57.5	1012.7	75.2	83.4	11.8	63.7	1012.0	63.3	73.7	8.2	64.7	1014.7
Long Island	54.3	64.3	7.7	66.0	1012.6	72.1	79.2	11.5	72.7	1012.0	61.7	70.8	7.1	71.2	1014.5
White Plains	55.2	68.0	6.9	61.2	1000.3	72.0	79.8	10.6	70.0	1000.0	60.2	70.7	6.7	70.1	1002.3
Hudson Valley-South	55.0	68.9	6.7	61.6	1003.2	70.9	78.9	10.4	72.5	1002.7	58.1	68.9	6.9	74.5	1005.2
Hudson Valley-North	54.3	67.9	6.7	58.8	1002.8	70.4	78.8	9.9	70.7	1002.2	56.6	67.4	7.3	74.4	1004.8
Adirondacks and North	49.5	62.3	4.1	63.0	991.0	66.4	75.1	6.8	72.6	989.9	52.8	64.8	4.5	75.4	992.4
Mohawk Valley	52.1	66.4	6.2	63.8	993.2	68.7	77.0	7.7	72.1	993.8	55.6	67.2	6.2	75.9	996.2
Binghamton	51.4	65.7	5.3	60.5	958.4	67.2	75.4	13.0	70.5	959.7	54.7	66.1	8.9	72.6	961.3
Rochester	51.8	65.6	6.8	63.7	996.3	68.8	77.1	9.6	72.0	995.7	55.9	67.7	7.0	75.5	997.9
Central Lakes	53.2	67.8	5.9	61.6	988.5	69.8	78.4	10.1	70.0	988.1	56.7	68.5	6.9	73.6	990.0
Western Plateau	51.7	66.1	6.4	60.8	962.0	67.2	75.3	11.0	71.4	963.2	54.4	66.2	6.7	74.4	964.7
Buffalo	52.0	65.8	8.9	62.4	983.6	69.4	77.5	12.0	69.1	983.5	57.2	68.5	9.2	72.2	985.2

Abbreviations: PM_2.5_: Particular matter less than 2.5 μm in aerodynamic diameter; RH: Relative humidity; BP: Barometric pressure. * The units of temperature, PM_2.5_, RH, and BP are Fahrenheit (°F), μg/m^3^ LC, %, and mb, respectively.

**Table 3:** Odds ratios for association between extremely hot days in lagged mean temperature and ED elderly visits by different extreme heat indicators for CVD in NYS, April–October 2005–2013.

Lag Days	90th	*p*-Value	95th	*p*-Value	97th	*p*-Value
Lag 0	0.991 (0.973–1.009)	0.3178	1.012 (0.998–1.027)	0.0891	1.014 (0.984–1.044)	0.3722
Lag 1	1.007 (0.989–1.025)	0.4634	0.997 (0.983–1.011)	0.6836	0.992 (0.963–1.022)	0.6002
Lag 2	0.999 (0.982–1.016)	0.9268	1.009 (0.995–1.023)	0.2087	0.999 (0.970–1.029)	0.9266
Lag 3	**1.018 (1.001–1.035)**	**0.0404**	1.006 (0.992–1.019)	0.3944	0.996 (0.967–1.025)	0.7788
Lag 4	**1.020 (1.003–1.037)**	**0.0245**	1.002 (0.989–1.016)	0.7491	1.015 (0.986–1.045)	0.3223
Lag 5	**1.025 (1.007–1.042)**	**0.005**	**1.023 (1.009–1.038)**	**0.001**	1.025 (0.995–1.055)	0.0983
Lag 6	**1.022 (1.005–1.039)**	**0.0103**	**1.013 (1.000–1.026)**	**0.0422**	**1.035 (1.008–1.063)**	**0.012**

**Table 4:** Association between extremely hot days ^a^ and ED elderly visits for CVD diseases in New York State, June–August 2005–2013, stratified by demographics and geographic region.

Demographic Variables	No. of Cases (%)	OR (CI) ^b^	Wald Test *p*-Value ^c^
Gender			0.0477
Female	89,961	1.004 (0.973–1.037)	0.7825
Male	87,871	0.031 (0.998–1.065)	0.0698
Age			0.8432
65–75	94,249	1.027 (0.995–1.061)	0.0992
76+	83,583	1.006 (0.974–1.039)	0.7165
Race			0.7125
White	130,380	1.022 (0.996–1.050)	0.1019
Black	21,719	0.951 (0.889–1.016)	0.1362
Others	25,733	1.046 (0.985–1.110)	0.1425
Ethnicity			0.1052
Hispanic	14,039	1.003 (0.924–1.088)	0.9439
Non-Hispanic	163,793	1.018 (0.994–1.043)	0.1354
Payment source			0.8326
Medicare	109,879	1.011 (0.982–1.041)	0.4765
Medicaid	4,009	1.121 (0.962–1.307)	0.1425
Other	63,944	1.024 (0.986–1.063)	0.2249
Region			0.0004
NYC—LaGuardia Airport	19,897	0.993 (0.929–1.063)	0.848
NYC—Kennedy Airport	35,162	1.040 (0.988–1.095)	0.1316
Staten Island	2,890	1.042 (0.871–1.246)	0.6525
Long Island	25,647	**1.085 (1.024–1.149)**	**0.0054**
Westchester	9,764	0.985 (0.894–1.085)	0.7595
Hudson Valley—South	6,970	0.946 (0.843–1.062)	0.3473
Hudson Valley—North	10,671	0.957 (0.873–1.049)	0.3469
Adirondacks and North	4,749	0.974 (0.852–1.115)	0.7056
Mohawk Valley	5,320	0.986 (0.849–1.144)	0.8484
Binghamton	9,916	1.059 (0.960–1.168)	0.2536
Rochester	9,404	0.953 (0.865–1.049)	0.3249
Central Lakes	9,000	0.944 (0.856–1.042)	0.2555
Western Plateau	4,517	1.013 (0.880–1.167)	0.8559
Buffalo	17,645	1.042 (0.975–1.115)	0.2226

Abbreviations: OR, odds ratio; CI, confidence interval. ^a^ Extremely hot day is defined as daily mean temperature >95th percentile of regional monthly mean temperature. ^b^ All the results had been adjusted for relative humidity, PM_2.5_ value and barometric pressure. ^c^ Wald Chi-square tests for homogeneity, the *p*-value for demographic variables indicate the statistical significance in each subgroup.

**Figure A1:**
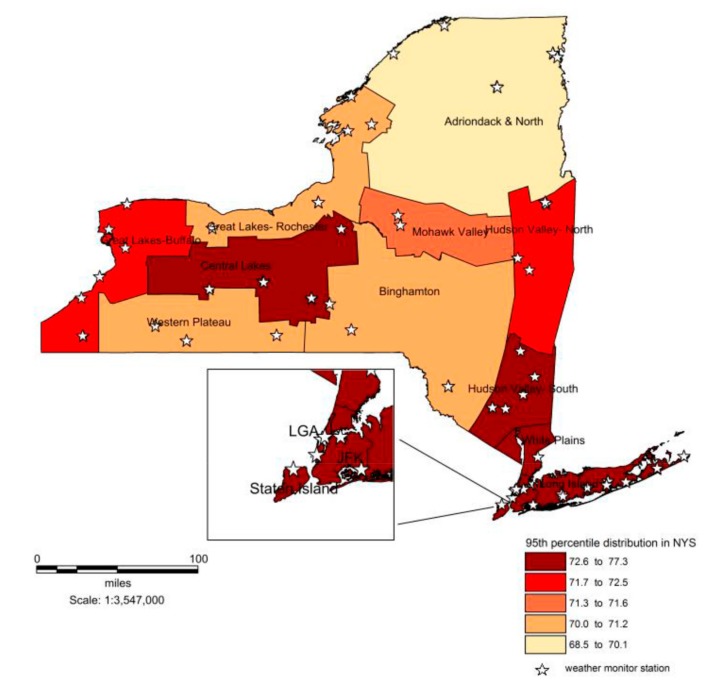
Map of the 95th percentile of daily mean temperatures over the study period in New York State, April–October 2005–2013.

## Appendix A

**Table A1:** The association between frequency of extremely hot days and elderly ED visits for cardiovascular diseases in New York State, April–October 2005–2013.

Extremely Hot Days Frequency	April–May	June–August	September–October
	Odds Ratio (CI) *	Odds Ratio (CI) *	Odds Ratio (CI) *
1	0.976 (0.964–0.989)	1.006 (0.997–1.015)	0.994 (0.984–1.004)
2	0.980 (0.961–1.000)	1.018 (1.009–1.028)	0.995 (0.983–1.008)
≥3	-	1.005 (0.959–1.053)	0.837 (0.764–0.918)

* The reference group is frequency of extremely hot days is 0. - means there is no eligible extremely hot days in this time period.

**Table A2:** The association between duration of extremely hot days and elderly ED visits for cardiovascular diseases in New York State, April–October 2005–2013.

Extremely Hot Days Duration	April–May	June–August	September–October
	Odds Ratio (CI) *	Odds Ratio (CI) *	Odds Ratio (CI) *
2	0.980 (0.965–0.995)	1.020 (1.009–1.030)	0.992 (0.977–1.007)
3	-	0.998 (0.937–1.062)	0.833 (0.756–0.917)

* reference group is consecutive extremely hot days is 0. - means there is no eligible extremely hot days in this time period.
